# Temperature-Responsive Polymer Microgel-Gold Nanorods Composite Particles: Physicochemical Characterization and Cytocompatibility

**DOI:** 10.3390/polym10010099

**Published:** 2018-01-20

**Authors:** Aslam Khan, Tajdar Husain Khan, Maqusood Ahamed, Ahmed Mohamed El-Toni, Ali Aldalbahi, Javed Alam, Tansir Ahamad

**Affiliations:** 1 King Abdullah Institute for Nanotechnology, King Saud University, Riyadh 11451, Saudi Arabia; mahamed@ksu.edu.sa (M.A.); aamohammad@ksu.edu.sa (A.M.E.-T.); aaldalbahi@ksu.edu.sa (A.A.); javaalam@ksu.edu.sa (J.A.); 2 College of Pharmacy, Prince Sattam Bin Abdulaziz University, Al-Kharj 11942, Saudi Arabia; d.khan@psau.edu.sa; 3 Central Metallurgical Research and Development Institute, CMRDI, Helwan 11421, Egypt; 4 Department of Chemistry, King Saud University, Riyadh 11451, Saudi Arabia; tahamed@ksu.edu.sa

**Keywords:** microgel, gold nanorods, temperature responsive, poly-*N*-isopropylacrylamide

## Abstract

In this paper, we report an easy route for preparing new metal nanorod-polymer composites consisting of gold nanorods, Au NRs, and temperature responsive copolymer “microgel” particles. The microgel particles of ~200 nm in size, which contain carboxylic acid groups, were prepared by surfactant-free emulsion polymerization of a selected mixture made of *N*-isopropylacylamide and acrylic acid in the presence of a cross-linker *N*,*N*′-methylenebisacrylamide. The electrostatic interactions between the cationic cetyltrimethylammonium bromide (CTAB) stabilized Au NRs and anionic microgel particles were expected to occur in order to prepare stable Au NRs-microgel composite particles. The optical and structural characterization of the composite was achieved using UV-Vis spectroscopy, Field emission scanning electron microscopy (FESEM), Transmission electron microscopy (TEM) and dynamic light scattering (DLS). TEM image shows that Au NRs are attached on the surface of the microgel particles. Dynamic light scattering measurements prove that the composite particles are temperature responsive, which means the particles undergo a decrease in size as the temperature increases above its phase transition temperature. In vitro cytotoxicity of the composite materials were tested by 3-(4,5-Dimethylthiazol-2-yl)-2,5-diphenyltetrazolium bromide (MTT), Lactate dehydrogenase (LDH), and hemolysis assay, which showed non-toxicity (biocompatibility).

## 1. Introduction

Stimuli-responsive polymers or so-called “smart polymers” have the capacity of altering their chemical or physical properties upon exposure to an external environment. Over the last two decades, these stimuli-responsive polymers have been studied broadly for their promising applications as biosensors [1,2] for their drug delivery [2,3,4,5] and for their use as regenerative medicine [6,7]. Among these stimuli-responsive polymers, poly(*N*-isopropylacrylamide) (PNIPAM) and its copolymers or microgels/nanogels (three dimensionally crosslink colloidal particles) have been an area of interest due to the fact that this material has the capacity to alter or respond to external stimuli [8]. These polymers show phase separation behavior or so-called “lower critical solution temperatures” (LCSTs). When the temperature is higher than its LCST, the polymer becomes hydrophobic (dehydrated/shrunken state) in water and the colloidal solution turns opaque [5]. Below their LCST, the same polymer is translucent in aqueous media and hydrophilic (i.e., hydrated/swollen state). At the LCSTs or phase separation temperatures, the cross-linked polymer chains undergo a volume phase transition (VPT)—The polymers expand below the LCST, and collapse above the LCST [3,5,9,10]. The phase transitions of these polymers are usually reversible upon the heating and cooling cycle. Because of this behaviour (phase transition) during the heating-cooling cycle, these polymers are of great scientific importance for fundamentally understanding the polymer phase transition phenomenon and its practical applications. Another feature that makes PNIPAM well-suited for application is the possibility to tune its transition temperature by co-polymerization with hydrophobic and hydrophilic segments, which results in a variation of the hydrophilic-hydrophobic balance of the polymer [5,11].

The gold nanoparticle (NP) is one of the most vital and interesting nanomaterials among metal nanoparticles, and has been shown to be a promising material due to its unique characteristics, such as surface plasmon resonance (SPR) [12,13,14,15]. Since Au NPs are reported to induce slight toxicity in some cases, it is preferable to modify them with a polymer [16] for significant uses in medical and clinical applications. Among the polymers used, stimuli-responsive polymers [16,17] are of great interest because their properties can be transformed and manipulated by changing the external environment. The thermo-responsive polymer/Au or silver NP composites have also been designed and developed to build stimuli-responsive therapeutic systems, with the capacity to combine the features of intelligent response, compatibility, and spatial and temporal control over the liberation of pharmacologically active compounds [16]. A different reported approach is to encapsulate Au NPs into PNIPAM gel, whereby Au NPs were coated with a thin layer of styrene-divinyl benzene followed by polymerization of NIPAM on the coated NPs. The swelling-deswelling transition of these particles showed a surface plasmon shift [18]. Budhalall et al. [19] developed Au NPs/NIPAM-*co*-Am hybrid core-shell microgels that can be actuated by visible light and microwave radiation or temperature. Incorporating Au NPs sped up the responsive kinetics of PNIPAM, hence enhancing the sensitivity of PNIPAM to external stimuli. Boyer et al. [20] designed and developed a series of PEG-based thermo-responsive polymer/Au hybrid NPs that have shown to have not only tunable thermo-responsive behavior, but also antifouling-protein resistance surface. Das et al. [21] prepared PNIPAM-*co*-maleic acid microgel particles covered with positively charged Au NRs and demonstrated swelling and de-swelling behavior using the laser-on and laser-off technique. Liang et al. [22] also made outstanding contributions to the cellular uptake of densely packed thioesters containing PNIPAM coated Au NPs. They found that positively or negatively charged Au NPs were taken up by the cells with greater efficiency than neutral Au NPs.

Among the gold nanoparticles, rod-shaped gold particles are one of the most interesting materials from an optical point of view. This is due to the gold nanorods exhibiting strong anisotropy, which shows two plasmon resonances called transverse and longitudinal plasmon resonance. The longitudinal SPR can easily be tuned to the near-infra red (NIR) region [15,23]. The position of the longitudinal SPR band mainly depends on the aspect ratio of the rods but can also be strongly affected by the dielectric environment and the juxtaposition of other nanorods [24]. The gold nanoparticles including Au NRs can carry and functionalize a number of small molecules for efficient drug delivery [23], DNA-based thermo-chemo therapy [25] and more. Furthermore, most of the Au NRs surfaces are covered and stabilized by surfactants such as cetyltrimethylammonium bromide (CTAB), which is crucial to maintaining the nanorod morphology [13,26,27,28]. The CTAB molecule alone is reported to be highly toxic to the cells at a sub-micro molar dose [29]. Recently, a study by Wan et al. [30] reported that surface modification or functionalization of Au NRs greatly influences the cytotoxicity and cellular uptake of the nanoparticles.

To date, several methodologies for preparing Au NRs with stimuli-responsive polymers have been synthesized [31] and investigated their properties. For example, Kozlovskaya et al. [32] reported incorporating Au NRs into the swollen cross-linked PMMA hydrogel films and studied their optical properties and swelling-deswelling behavior based on pH changes. Duan et al. [33] developed a method for preparing Au NRs coated with chitosan derivatives by further conjugating the compounds with doxorubicin (anti-cancer drug) and studying their photothermal effect. Kawano et al. [34] have prepared a well-defined core-shell structure composed of silica coated Au NRs with PNIPAM. The photothermal phase transition and accumulation of Au NRs in mice organs using the near-IR laser was studied. Our research group has also reported [35] earlier on the development of thermo and pH-responsive microgel-based Au NRs and their structural characterization with atomic force microscopy and the volume phase transition behavior upon near-IR laser irradiation. There is still a challenging task to employ CTAB molecules currently used as a capping agent on the Au NRs surface because of the well-known toxicity characteristics of CTAB shown to induce toxicity to cells for a wide range of biological applications [29,36,37,38,39]. Although numerous research publications have appeared in the literature that attempt to synthesize Au NRs by avoiding CTAB [40], they have all been unsuccessful. For therapeutic applications, it is very important to either completely remove CTAB by surface modification, or to protect the CTAB layer present on the surface of the NRs that can render both intrinsic and extrinsic benefits to the nanoparticles to improve their efficacy in biomedicine. Taking into account the points above, it is very important to develop a new strategy to prepare less toxic Au NRs or its composites by means of a simple and versatile process for future biomedical uses. To the best of our knowledge, only few reports are currently available that provide a simple synthetic approach to PNIPAM-based Au NRs-microgel composites and their cytotoxicity studies.

In this paper, we report the results obtained by studying the properties of the temperature responsive Au NRs-microgel composite particles prepared by electrostatic interactions between the two opposite-charged particles where the NRs are connected to the microgel particles. The as-prepared composite particles in an aqueous solution were studied using dynamic light scattering (DLS) and found that the sizes are greatly influenced by the temperature without disturbing the thermo-sensitivity of the gel network. The method for preparing the composite materials is simple, versatile, and stable for months. Moreover, the as-prepared composite particles showed no sign of toxicity in in vitro hemolysis test as well as MTT and LDH assays. All these properties make these prepared composite materials more beneficial for further biological applications like photothermal-therapy and drug delivery application.

## 2. Materials and Methods

### 2.1. Materials

The *N*-isopropylacrylamide was purchased from Aldrich, St. Louis, MO, USA, purified with hexane, and dried under vacuum before use. *N*,*N*′-methylenebisacrylamide (Aldrich, St. Louis, MO, USA), acrylic acid (Aldrich, St. Louis, MO, USA), ammonium persulfate (Merck, Darmstadt, Germany), CTAB (Aldrich, St. Louis, MO, USA), AgNO_3_ (Merck, Darmstadt, Germany), ascorbic acid (Aldrich, St. Louis, MO, USA), and HAuCl_4_ (Sigma-Aldrich, St. Louis, MO, USA) were all used as-received. Milli Q water was used in all reactions solution preparations.

### 2.2. Experiment

The *N*-isopropylacrylamide-*co*-acrylic acid microgel particles were prepared by the surfactant-free emulsion polymerization (SFEP) method previously described [41]. Briefly, in a 100 mL RB, add 50 mL distilled water, 0.1698 g NIPAM, 0.0117 g (5% of the monomer) MBA, 0.062 g of AAc were dissolved by magnetic stirring and degassed by bubbling N_2_ for half an hour. The temperature was raised to 70 °C and the polymerization was initiated with 150 µL (0.01 M in water) of ammonium persulfate initiator. After 10–15 min the colorless solution turned cloudy, which indicated that the polymerization reaction was initiated. The reaction should proceed for 3 h. The substance was cooled to room temperature, centrifuged at 12,000 rpm by elevating the temperature at 40 °C, collected, and washed with distilled water. This process was repeated five times to remove unreacted monomers present. Finally, the microgels were purified by dialysis against distilled water then freeze-dried (EYELA FDU-2100, Tokyo, Japan) for further study.

Secondly, Au NRs with a CTAB surfactant coating were synthesized individually using the seed-growth method [42]. First, we synthesized 3-nm-sized seed Au nanoparticles stabilized with CTAB. Then nanorods were prepared by adding nanoparticle seed solution (6 μL) to the growth solution: an aqueous mixture of CTAB (40 mL, 0.01 M), AgNO_3_ (0.4 mL, 0.01 M), HAuCl_4_ (2 mL, 0.01 M), and ascorbic acid (0.32 mL, 0.1 M). The pH of the solution was adjusted to 1.5 by adding 0.1 M HCl. The resultant solution was gently stirred for 20 s and left undisturbed overnight. The solutions were centrifuged for 30 min at 12,000 rpm, and the supernatant was discarded. The ppt was re-suspended in water and the same centrifugation process was repeated three times.

The Au NRs-microgel composites were prepared as follows: 6 mL of as-prepared Au NRs solution (0.73 mg of Au/mL) was added drop-by-drop to a vial containing 40 mL of the microgel particles maintained at about pH 9 (2 mg of microgels in 98 mL of water) and vigorously stirred for 48 h at ambient temperature. The mixture containing the microgel-nanorods, Au-NRs-microgel was purified by centrifugation to remove excess unbounded CTAB and then re-dispersed in 6 mL of deionized water for further study.

### 2.3. Cell Viability Assay

MTT Assay: Human breast cancer cell lines MCF-7 were used in this study. Cells were cultured in DMEM/F-12 medium supplemented with 10% FBS and 100 U/mL penicillin-streptomycin at 5% CO_2_; 37 °C. Cell viability was measured by the MTT assay as reported in our previous publication [43,44]. Briefly, 1 × 10^4^ cells per well were seeded in a 96-well plates and the samples were exposed to different concentrations of 0, 5, 10, 25, 50 and 100 µg/mL for 24 h. After the exposure was completed, the medium was removed from each well and replaced with new medium containing MTT solutions, and finally incubated for 3 h at 37 °C until a purple color was formed. The absorbance was measured at 570 by using a microplate reader (Synergy-HT, Biotek, Winooski, VT, USA).

Lactate dehydrogenase (LDH) Assay: An LDH assay was carried out using a BioVision LDH-cytotoxicity colorimetric assay kit according to the manufacturer’s protocol. The protocol was followed in a similar way to the MTT assay mentioned above. Finally, after incubation, the absorbance was read at 340. LDH levels in the media vs the cells were quantified and compared to the control value as per the instruction given in the kit.

### 2.4. Cell Hemolysis on Blood Agar

Cell hemolysis on blood agar plates were performed [45,46]. Fresh whole blood was collected by a retro orbital sinus puncture under light anesthesia from healthy mice and transferred into heparinize tubes to prevent coagulation. The blood agar plates were prepared by adding blood (5%) to blood agar base. 4 g of Tryptic Soy Blood Agar Base (TSBA) was taken, suspended into the 100 mL of distilled water, allowed to mic thoroughly, and autoclaved at 121 °C for 15 min. The autoclaved TSBA was allowed to cool to 45–50 °C and then 5 mL of sterile defibrinated blood was aseptically added. The substance was mixed thoroughly. The petri-dishes (6-well) were arranged onto the clean safety hood and then the warm blood agar was gently poured onto the plates. Sterile samples were added in the center of each gel plate (where agar was not solid) and brought into contact with Eagle’s minimum essential medium (MEM) culture medium under sterile condition. The gel plate was incubated at 37 °C using the cell culture oven. The plates were then examined every 12 h for the zone of hemolysis. The surface around each hydrogel sample was observed and photographed after 12, 18, and 36 h by a digital Cannon PowerShot camera. The images were compared to the control (a blood gel which had been in contact with any sample).

### 2.5. Measurements

The ^1^H NMR spectra of the microgel was studied using a VARIAN 400 spectrometer (400 MHz). Transmission electron microscopy (TEM) images were obtained using a JEOL (Tokyo, Japan) 2100 microscope operated at 200 kV. Field emission scanning electron microscopy (FESEM) studies were performed on a JSM 7600 F microscope (JEOL, Tokyo, Japan) at 5 kV. UV-Vis spectra of the samples were recorded using a Shimadzu (Tokyo, Japan) 2550 spectrophotometer. Finally, the size of the composite particles dispersed in water (20 mg/mL) was determined by Malvern Nano ZS dynamic light scattering (DLS) varying from 20 to 50 °C.

## 3. Results and Discussion

**Figure 1:**
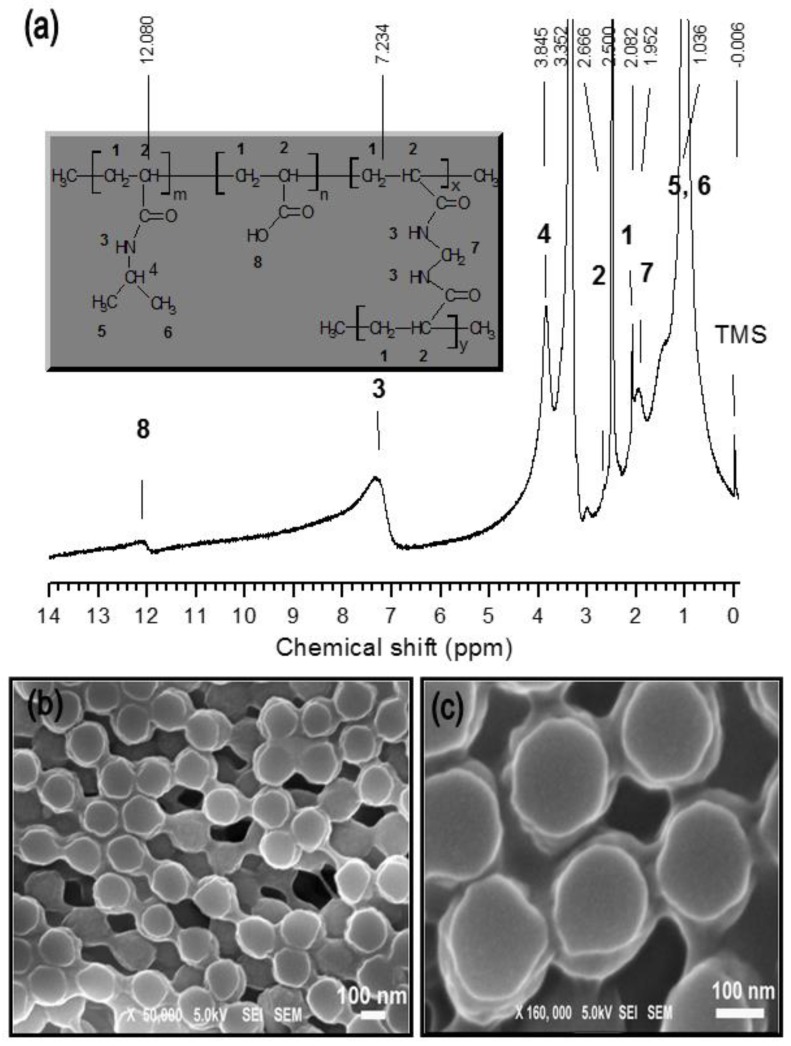
(**a**) is the ^1^H NMR spectra of the poly(NIPAAm-*co*-AAc) microgel particles. Instet figure is the chemical structures of the as-synthesized microgel; (**b**,**c**) Field emmision scanning electron microscopy images of microgel particles in two different magnifications (magnification = 50,000× and 160,000×, respectively).

The copolymer poly(*N*-isopropylacrylamide-*co*-acrylic acid) [poly(NIPAAm-*co*-AAc)] microgels with a hydrodynamic diameter of ca. 200 nm were synthesized by surfactant-free emulsion polymerization. The copolymer products were characterized by NMR. The ^1^H NMR spectra of the copolymer is shown in Figure 1a. The typical proton signals around 1.0 ppm ascribed to the –CH_3_ protons of the NIPAM units were observed in the ^1^H NMR spectra of the copolymers, and the signal from 1.9 to 2.6 ppm ppm was caused by the polymer backbone assigned to the (–CH_2_–CH–) protons. The peak at 12.0 ppm was attributed to the (–COOH) protons of the acrylic acid groups present in the copolymer.

Figure 1b,c shows the FESEM images of as-prepared microgel particles via SFEP method, drop cast from 5 µL of solution on a glass slide and air dried. It reveals that the blobs of smooth surface, spherical shape particles of approximately 200 ± 20 nm in diameter are interconnected to one another.

**Figure 2:**
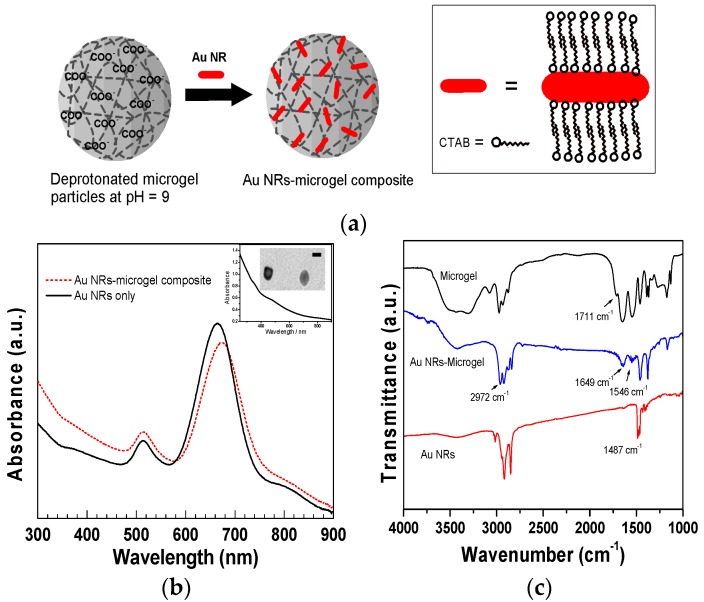
(**a**) Scheme illustrating the formation of Au NRs-microgel composite particles using electrostatic interactions between the cationic charge carrying Au NRs and anionic microgel particles; (**b**) UV-Vis spectra of gold NRs prior to (solid line) and following NR incorporation in microgels i.e., composite particles (dash line). Inset is the absorbance peak and TEM image of seed solution. Scale bar is 5 nm; (**c**) FTIR spectra of the microgel, Au NRs and Au NRs-microgel composite.

The preparation of the Au NRs-microgel composite colloids was based on electrostatic interactions between the swollen, highly hydrated, anionic (acrylic acid containing) microgel particles and cationic surfactant (CTAB) stabilized Au NRs. pH of the as-synthesized microgel solution was adjusted to pH 9 so that a proton is removed in the carboxyl groups present in the microgel particles. At pH 9, the polymer is highly swollen due to osmotic effect and electrostatic repulsion between the AAc residues, which both favour incorporating the positively charged Au NRs into the negatively charged voids of microgel particles through electrostatic interactions. A schematic diagram of the formation of Au NRs-microgel composite particles is shown in Figure 2a. The UV-Vis absorption spectra of the as-prepared Au NRs and the Au NRs-microgel composites are also shown in Figure 2b. The absorbance peak at 514 nm corresponds to the transverse plasmon oscillations and the peak with higher intensity at 689 nm corresponds to the longitudinal plasmon resonance peak [47]. In Figure 2b, the plasmon band of the Au NRs embedded in the microgels is centered at 697 nm, which means it was shifted by 8 nm of the longitudinal SPR when compared with the band of bar Au NRs (without microgels) dispersed in the water solution. However, we did not notice any shift of the transverse peak. The red-shift presumably originated from a minor end-to-end aggregation of Au NRs, causing the effective elongation and the change in the dielectric constant of the environment surrounding the Au NRs [48].

To further demonstrate the successful encapsulation of Au NRs, the microgel/Au NRs were characterized by FTIR (Figure 2c). The as-prepared Au NRs exhibited a characteristic IR absorbance at 1487 cm^−1^ (δ_asym_, C–H), from the CH_3_–N^+^ moiety of CTAB (Figure 2c) [49]. In the IR spectra, the main characteristic peak assignments are located at 2972 cm^−1^ (CH_3_ asymmetric stretching), 1649 cm^−1^ (C=O stretching, amide I), and 1546 cm^−1^ (N–H bending, amide II), [5] indicating that this was the main contribution of pNIPAAm to Au NRs-microgel composite. In addition to these peaks, the carbonyl stretching bond attributed to the carboxylic acid group of AAc units is observed at 1711 cm^−1^ in the microgel sample (Figure 2c), but the same peak disappeared in the Au NRs-microgel composite sample, which supports the UV-Vis results. Notably, the contribution from the cross-linker MBA in the FTIR spectra may be obscured by the pNIPAAm since the former is present in a low amount and has a chemical structure similar to NIPAAm.

**Figure 3:**
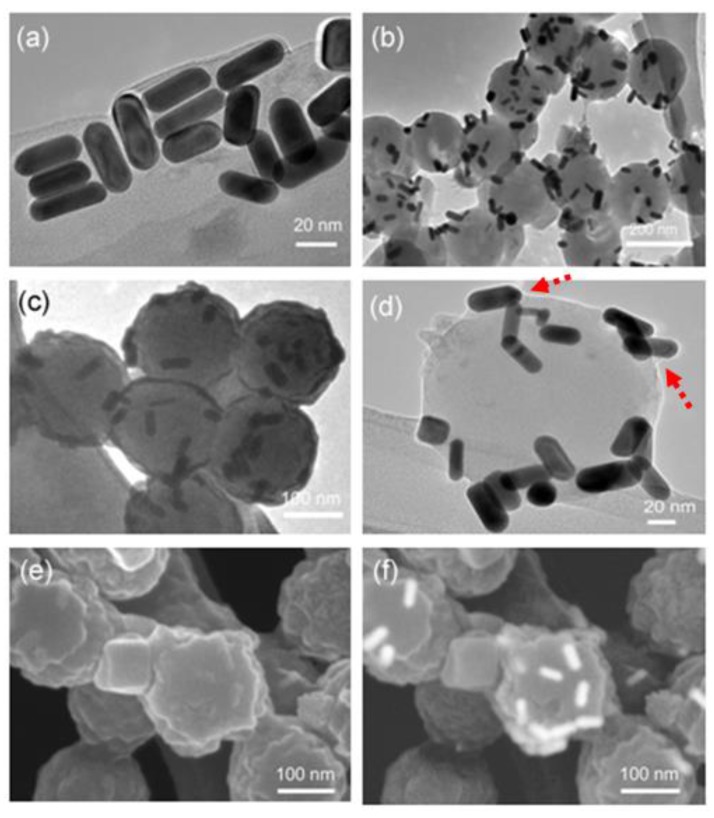
Transmission electron microscopy image of (**a**) Au NRs synthesized by seed-mediated method, (**b**–**d**) as-prepared Au NRs-microgel composite particles. Field emission scanning electron micrographs of Au NRs-microgel composite particles (**e**) using secondary and (**f**) back-scattered mode. (magnification = 200,000×).

TEM image of Au NRs prepared by seed-mediated method is shown in Figure 3a. The Au NRs have an average length, width, and aspect ratio of 48 ± 2.1 nm by 17 ± 1.8 nm by 2.8 ± 1.1, respectively. The TEM images (Figure 3b,c) of as-prepared Au NRs-microgel composite show that most of the NRs (rod-shaped and dark color substance) are located on the surface of the microgel particles, and no noticeable free NRs are observed. The figures mentioned above illustrate that the morphology of the microgel particles are spherical and secondly, that the microgels have a smooth surface since it can also be observed in the enlarged image shown in Figure 3d. It can be clearly seen from Figure 3d that a nimbus or a thin opaque layer of the polymer enveloped (cf. red color arrows) the Au-NRs. The morphology of the Au NRs-microgel composite particles was further observed under FESEM in a secondary electron (SE) and back-scattered electron (BSE) mode (cf. Figure 3e,f). It reveals that the Au NRs (a rough surface with bumps of white colored spots) were evenly distributed and attached onto the surface of the microgel particles when observed under SE mode. A clearly visible Au NRs in the microgel matrix is visible when observed using BSE mode. 

**Figure 4:**
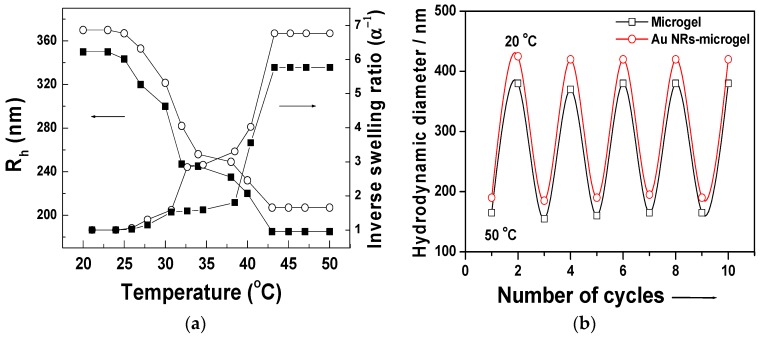
(**a**) Temperature vs hydrodynamic size dependence and inverse swelling ratios plot of the microgel particles (squares) and Au NRs-microgel composite particles (circles) dispersed in water measured by dynamic light scattering; (**b**) Changes of hydrodynamic size showing reversible upon heating and cooling cycles between 20 and 50 °C.

The temperature-responsive swelling properties of the composite particle were studied using DLS experiments. As expected, there is a significant change in size induced by the change in the temperature. As shown in Figure 4a, at 20 °C, the particle size is 370 nm, and at 33 °C, the particle size is reduced to 248 nm due to the collapse of the poly-NIPAAm chains. As the temperature increased, a sharp change in the particle size was observed and the final hydrodynamic size was 220 nm at 50 °C. The inverse swelling ratio α^−1^ of the microgel particle with or without Au NRs is also shown in Figure 4a. The swelling ratio (α) was defined as the ratio between the particle volume in the shrunken (*V*_shrunken_) and the swollen (*V*_swollen_) state. Comparing the relative swelling volume shows that both components behave very similarly. No significant difference in swelling behavior from the pure microgel particles was observed. The as-prepared colloidal solution of Au NRs-microgel composites were highly stable and did not show any sign of aggregation of precipitation even after storage for several months at ambient temperature. Figure 4b shows that the volume phase transition of these Au NRs-microgel composites is completely reversible over multiple cycles of heating and cooling, which clarifies the robust nature of the stimuli-responsive characteristics of the polymer and its composites.

**Figure 5:**
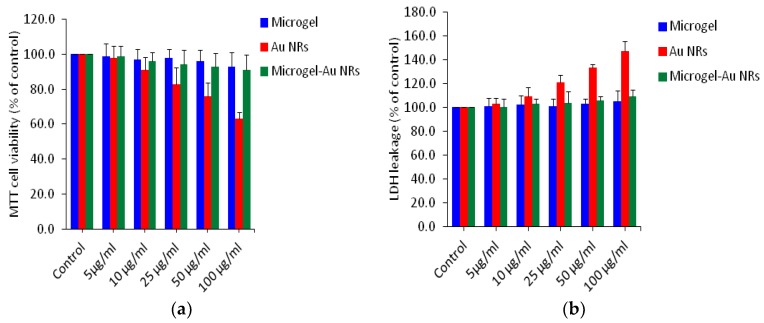
Cytotoxicity of microgel, Au NRs and Au NRs-microgel in MCF-7 cells. Cells were treated with different concentrations of (5–100 µg/mL) our samples for 24 h. (**a**) cell viability assay and (**b**) cell membrane damage (LDH assay). Data represented are mean ± SD of three identical experiments (*n* = 3) made in three replicate.

The MTT assay evaluates the mitochondrial function by measuring the ability of viable cells to reduce MTT into a blue formazon product [43]. LDH is an enzyme extensively present in cytosol that alters lactate to pyruvate. When plasma membrane integrity is disordered, LDH leaks into culture media and the extracellular level is raised [50]. We utilized human breast cancer cells (MCF-7) to examine the cytotoxicity of our samples using MTT and LDH assay at the concentrations of 0, 5, 10, 25, 50 and 100 µg/mL for 24 h (Figure 5a,b). We examined more than one assay to evaluate the cytotoxicity of the nanoparticles to get more reliable data. The above two assays have shown that microgels (without Au NRs) and Au NRs-microgel composites in all concentrations tested did not produce significant cytotoxicity. In the case of bar Au NRs, as the concentration of the nanoparticles increased to 10, 25, 50 and 100 µg/mL, cytotoxicity was observed in a dose-dependent manner. In the MTT assay, cell viability was significantly decreased to 84%, 78%, and 62% for concentrations at 25, 50 and 100 µg/mL, respectively (Figure 5a). Results published in scientific literature concerning toxicity of CTAB and Au NRs-capped with CTAB (so called bar Au NRs) were consistent. These materials have been shown to generate toxicity [51,52]. LDH assays, which identify the release of LDH from the cytoplasm as a response to cell membrane impairment, have often been used to understand the effect of attaching Au NRs to the surface of the cell membrane [53]. Wang and other research groups have reported that bar Au-NRs induce more LDH release than other surface-modified Au-NRs or free CTAB molecules [51,52] This prompted us to investigate the toxicity of Au NRs and Au NRs-microgel composites, which have tremendous potential for biological application. Figure 5b shows the results of cell viability obtained by LDH leakage (membrane integrity) of Au NRs, microgels, and Au NRs-microgel samples. Interestingly, in both the assays, all the concentrations we examined show no significant cytotoxicity to the Au NRs-microgel samples when exposed to MCF-7 cells. Our results are in agreement with those reported previously by other researchers that poly(styrene sulfonated) modified [54] or PEGylated [55] Au NRs were shown to lessen the toxicity of the Au NRs and free CTAB on the given cell lines. The LDH assay results support the MTT data (as shown in Figure 5b).

**Figure 6:**
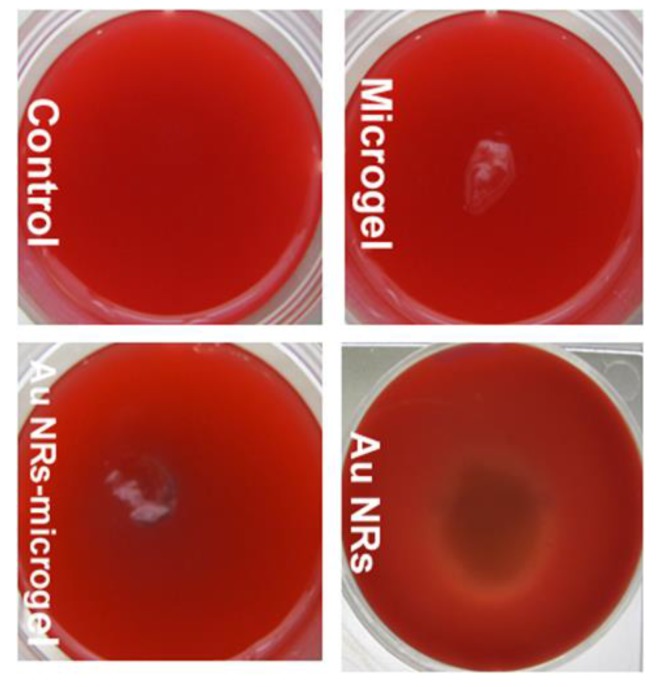
Results of cytotoxicity test by hemolysis with blood-agar using microgel, gold nanorods and Au NRs-microgel composite samples, after 18 h at 37 °C and a 5% CO_2_ flow in the oven for cell culture.

Furthermore, hemolytic activity was carried out by using the blood agar plate method. Hemolysins are enzymes that lyse red blood cells and are formed after cell deterioration [45,46]. The bacteria creating these enzymes express a whitish or translucent nimbus due to the existence of colonies causing from the lysis of red blood cells. In order to evaluate biocompatibility of the composite material developed herein, an in vitro hemolysis assay was performed on blood-agar media, incubated at 37 °C for 18 h and observed in a hemolysis zone. A cytotoxicity test using blood-rich agar allowed the verification of the degradation resulting from the action of the cytotoxic components present in a given substrate [56]. In our experiment, we have tested the microgel (lyophilized flaps), gold nanorods (dissolved in water), and Au NRs-microgel composite (lyophilized flaps). Figure 6 shows that a whitish or transparent halo around the gold nanorods sample was developed, which indicates that lysis of red blood cells occurred after 18 h. From this result it can be concluded that the gold nanorods coated with CTAB are toxic. The reason for this may be due to the unbounded (free) CTAB molecules present in the sample. These molecules may cause toxicity to the materials. This finding supports previous assertions that free CTAB may contribute to the cytotoxicity of CTAB-capped gold nanorods [29,57], even after two cycles of centrifugation and washing with water to remove unbounded CTAB molecules to the gold nanorods. However, the microgel alone and Au NRs-microgel composite samples did not show any hemolysis effect (no sign of transparent or white halo around the samples). Thus, this material seems to be non-toxic and could be useful for further biological applications. 

## 4. Conclusions

This discussion centers on a versatile and easy method for the assembly of gold nanorods onto the microgel particles based on electrostatic interactions. This method includes the introduction of carboxylic groups and their subsequent deprotonation of microgel particles and then uptake of cationic Au NRs through the presence of attractive Coulomb interactions in a solution. The as-prepared composite particles are temperature-responsive and undergo a decrease in particle size as the temperature increases above the phase transition temperature. The as-prepared Au NRs-microgel composite samples were stable for months when stored at ambient temperatures. When using in vitro studies, the Au NRs-microgel composite particles show no toxicity to the given cell lines. Therefore, it could find important applications in both drug delivery and photothermal therapies.

## References

[1] Wei M., Gao Y., Li X., Serpe M.J. (2017). Stimuli-responsive polymers and their applications. Polym. Chem..

[2] Dai S., Ravi P., Tam K.C. (2008). pH-Responsive polymers: Synthesis, properties and applications. Soft Matter.

[3] Strong L.E., West J.L. (2011). Thermally responsive polymer-nanoparticle composites for biomedical applications. Wiley Interdiscip. Rev. Nanomed. Nanobiotechnol..

[4] Dong L.-C., Hoffman A.S. (1990). Synthesis and application of thermally reversible heterogels for drug delivery. J. Control. Release.

[5] Khan A. (2007). Preparation and characterization of *N*-isopropylacrylamide/acrylic acid copolymer core-shell microgel particles. J. Colloid Interface Sci..

[6] Hoffman A.S. (2013). Stimuli-responsive polymers: Biomedical applications and challenges for clinical translation. Adv. Drug Deliv. Rev..

[7] MacEwan S.R., Chilkoti A. (2010). Elastin-like polypeptides: Biomedical applications of tunable biopolymers. Pept. Sci..

[8] Maeda T., Takenouchi M., Yamamoto K., Aoyagi T. (2009). Coil-Globule Transition and/or Coacervation of Temperature and pH Dual-Responsive Carboxylated Poly(*N*-isopropylacrylamide). Polym. J..

[9] Lai E., Wang Y., Wei Y., Li G., Ma G. (2016). Covalent immobilization of trypsin onto thermo-sensitive poly(*N*-isopropylacrylamide-*co*-acrylic acid) microspheres with high activity and stability. J. Appl. Polym. Sci..

[10] Sershen S.R., Westcott S.L., Halas N.J., West J.L. (2000). Temperature-sensitive polymer–nanoshell composites for photothermally modulated drug delivery. J. Biomed. Mater. Res..

[11] Lu Y., Mei Y., Drechsler M., Ballauff M. (2006). Thermosensitive Core-Shell Particles as Carriers for Ag Nanoparticles: Modulating the Catalytic Activity by a Phase Transition in Networks. Angew. Chem. Int. Ed..

[12] Huff T.B., Tong L., Zhao Y., Hansen M.N., Cheng J.-X., Wei A. (2007). Hyperthermic effects of gold nanorods on tumor cells. Nanomedicine (London).

[13] Ye T., Dai Z., Mei F., Zhang X., Zhou Y., Xu J., Wu W., Xiao X., Jiang C. (2016). Synthesis and optical properties of gold nanorods with controllable morphology. J. Phys. Condens. Matter.

[14] Nehl C.L., Liao H., Hafner J.H. (2006). Optical Properties of Star-Shaped Gold Nanoparticles. Nano Lett..

[15] Zhao T., Shen X., Li L., Guan Z., Gao N., Yuan P., Yao S.Q., Xu Q.-H., Xu G.Q. (2012). Gold nanorods as dual photo-sensitizing and imaging agents for two-photon photodynamic therapy. Nanoscale.

[16] Li D., He Q., Li J. (2009). Smart core/shell nanocomposites: Intelligent polymers modified gold nanoparticles. Adv. Colloid Interface Sci..

[17] Lee S.-M., Nguyen S.T. (2013). Smart Nanoscale Drug Delivery Platforms from Stimuli-Responsive Polymers and Liposomes. Macroolecules.

[18] Contreras-Cáceres R., Sánchez-Iglesias A., Karg M., Pastoriza-Santos I., Pérez-Juste J., Pacifico J., Hellweg T., Fernández-Barbero A., Liz-Marzán L.M. (2008). Encapsulation and Growth of Gold Nanoparticles in Thermoresponsive Microgels. Adv. Mater..

[19] Budhlall B.M., Marquez M., Velev O.D. (2008). Microwave, Photo- and Thermally Responsive PNIPAm-Gold Nanoparticle Microgels. Langmuir.

[20] Boyer C., Whittaker M.R., Luzon M., Davis T.P. (2009). Design and Synthesis of Dual Thermoresponsive and Antifouling Hybrid Polymer/Gold Nanoparticles. Macromolecules.

[21] Das M., Sanson N., Fava D., Kumacheva E. (2007). Microgels Loaded with Gold Nanorods:  Photothermally Triggered Volume Transitions under Physiological Conditions. Langmuir.

[22] Liang M., Lin I.-C., Whittaker M.R., Minchin R.F., Monteiro M.J., Toth I. (2010). Cellular Uptake of Densely Packed Polymer Coatings on Gold Nanoparticles. ACS Nano.

[23] Nam J., Won N., Bang J., Jin H., Park J., Jung S., Jung S., Park Y., Kim S. (2013). Surface engineering of inorganic nanoparticles for imaging and therapy. Adv. Drug Deliv. Rev..

[24] Ma W., Kuang H., Xu L., Ding L., Xu C., Wang L., Kotov N.A. (2013). Attomolar DNA detection with chiral nanorod assemblies. Nat. Commun..

[25] Song J., Im K., Hwang S.S., Hur J., Nam J., Ahn G.-O., Hwang S.S., Kim S., Park N. (2015). DNA hydrogel delivery vehicle for light-triggered and synergistic cancer therapy. Nanoscale.

[26] Gordel M., Piela K., Kołkowski R., Koźlecki T., Buckle M., Samoć M. (2015). End-to-end self-assembly of gold nanorods in isopropanol solution: Experimental and theoretical studies. J. Nanopart. Res..

[27] Heitsch A.T., Smith D.K., Patel R.N., Ress D., Korgel B.A. (2008). Multifunctional particles: Magnetic nanocrystals and gold nanorods coated with fluorescent dye-doped silica shells. J. Solid State Chem..

[28] Fernandez-Lopez C., Polavarapu L., Solis D.M., Taboada J.M., Obelleiro F., Contreras-Caceres R., Pastoriza-Santos I., Perez-Juste J. (2015). Gold Nanorod-pNIPAM Hybrids with Reversible Plasmon Coupling: Synthesis, Modeling, and SERS Properties. ACS Appl. Mater. Interfaces.

[29] Alkilany A.M., Nagaria P.K., Hexel C.R., Shaw T.J., Murphy C.J., Wyatt M.D. (2009). Cellular Uptake and Cytotoxicity of Gold Nanorods: Molecular Origin of Cytotoxicity and Surface Effects. Small.

[30] Wan J., Wang J.-H., Liu T., Xie Z., Yu X.-F., Li W. (2015). Surface chemistry but not aspect ratio mediates the biological toxicity of gold nanorods in vitro and in vivo. Sci. Rep..

[31] Choi R., Yang J., Choi J., Lim E.-K., Kim E., Suh J.-S., Huh Y.-M., Haam S. (2010). Thiolated Dextran-Coated Gold Nanorods for Photothermal Ablation of Inflammatory Macrophages. Langmuir.

[32] Kozlovskaya V., Kharlampieva E., Khanal B.P., Manna P., Zubarev E.R., Tsukruk V.V. (2008). Ultrathin layer-by-layer hydrogels with incorporated gold nanorods as pH-sensitive optical materials. Chem. Mater..

[33] Duan R., Zhou Z., Su G., Liu L., Guan M., Du B., Zhang Q. (2014). Chitosan-coated Gold Nanorods for Cancer Therapy Combining Chemical and Photothermal Effects. Macromol. Biosci..

[34] Kawano T., Niidome Y., Mori T., Katayama Y., Niidome T. (2009). PNIPAM Gel-Coated Gold Nanorods for Targeted Delivery Responding to a Near-Infrared Laser. Bioconjug. Chem..

[35] Khan A., Alhoshan M. (2013). Preparation and characterization of pH-responsive and thermoresponsive hybrid microgel particles with gold nanorods. J. Polym. Sci. A.

[36] Murphy C.J., Gole A.M., Stone J.W., Sisco P.N., Alkilany A.M., Goldsmith E.C., Baxter S.C. (2008). Gold Nanoparticles in Biology: Beyond Toxicity to Cellular Imaging. Acc. Chem. Res..

[37] Alkilany A.M., Murphy C.J. (2010). Toxicity and cellular uptake of gold nanoparticles: What we have learned so far?. J. Nanopart. Res..

[38] Alkilany A.M., Shatanawi A., Kurtz T., Caldwell R.B., Caldwell R.W. (2012). Toxicity and cellular uptake of gold nanorods in vascular endothelium and smooth muscles of isolated rat blood vessel: Importance of surface modification. Small.

[39] Grabinski C., Schaeublin N., Wijaya A., D’Couto H., Baxamusa S.H., Hamad-Schifferli K., Hussain S.M. (2011). Effect of Gold Nanorod Surface Chemistry on Cellular Response. ACS Nano.

[40] Sebastian V., Lee S.-K., Zhou C., Kraus M.F., Fujimoto J.G., Jensen K.F. (2012). One-step continuous synthesis of biocompatible gold nanorods for optical coherence tomography. Chem. Commun..

[41] Khan A., El-Toni A.M., Alrokayan S., Alsalhi M., Alhoshan M., Aldwayyan A.S. (2011). Microwave-assisted synthesis of silver nanoparticles using poly-*N*-isopropylacrylamide/acrylic acid microgel particles. Colloids Surf. A.

[42] Nikoobakht B., El-Sayed M.A. (2003). Preparation and Growth Mechanism of Gold Nanorods (NRs) Using Seed-Mediated Growth Method. Chem. Mater..

[43] Mosmann T. (1983). Rapid colorimetric assay for cellular growth and survival: Application to proliferation and cytotoxicity assays. J. Immunol. Methods.

[44] Ahamed M., Akhtar M.J., Siddiqui M.A., Ahmad J., Musarrat J., Al-Khedhairy A.A., AlSalhi M.S., Alrokayan S.A. (2011). Oxidative stress mediated apoptosis induced by nickel ferrite nanoparticles in cultured A549 cells. Toxicology.

[45] Coronado R., Pekerar S., Lorenzo A.T., Sabino M.A. (2011). Characterization of thermo-sensitive hydrogels based on poly(*N*-isopropylacrylamide)/hyaluronic acid. Polym. Bull..

[46] Hanks C.T., Wataha J.C., Sun Z. (1996). In vitro models of biocompatibility: A review. Dent. Mater..

[47] Huang X., Neretina S., El-Sayed M.A. (2009). Gold Nanorods: From Synthesis and Properties to Biological and Biomedical Applications. Adv. Mater..

[48] Shiotani A., Akiyama Y., Kawano T., Niidome Y., Mori T., Katayama Y., Niidome T. (2010). Active Accumulation of Gold Nanorods in Tumor in Response to Near-Infrared Laser Irradiation. Bioconjug. Chem..

[49] Nikoobakht B., El-Sayed M.A. (2001). Evidence for Bilayer Assembly of Cationic Surfactants on the Surface of Gold Nanorods. Langmuir.

[50] Wróblewski F., Ladue J.S. (1955). Lactic Dehydrogenase Activity in Blood. Proc. Soc. Exp. Biol. Med..

[51] Wang L., Jiang X., Ji Y., Bai R., Zhao Y., Wu X., Chen C. (2013). Surface chemistry of gold nanorods: Origin of cell membrane damage and cytotoxicity. Nanoscale.

[52] Wang L., Liu Y., Li W., Jiang X., Ji Y., Wu X., Xu L., Qiu Y., Zhao K., Wei T. (2011). Selective Targeting of Gold Nanorods at the Mitochondria of Cancer Cells: Implications for Cancer Therapy. Nano Lett..

[53] Indrasekara A.S.D.S., Wadams R.C., Fabris L. (2014). Ligand Exchange on Gold Nanorods: Going Back to the Future. Part. Part. Syst. Charact..

[54] Leonov A.P., Zheng J., Clogston J.D., Stern S.T., Patri A.K., Wei A. (2008). Detoxification of Gold Nanorods By Treatment With Polystyrenesulfonate. ACS Nano.

[55] Ali M.R.K., Rahman M.A., Wu Y., Han T., Peng X., Mackey M.A., Wang D., Shin H.J., Chen Z.G., Xiao H. (2017). Efficacy, long-term toxicity, and mechanistic studies of gold nanorods photothermal therapy of cancer in xenograft mice. Proc. Natl. Acad. Sci. USA.

[56] Fischer D., Li Y., Ahlemeyer B., Krieglstein J., Kissel T. (2003). In vitro cytotoxicity testing of polycations: Influence of polymer structure on cell viability and hemolysis. Biomaterials.

[57] Vigderman L., Manna P., Zubarev E.R. (2012). Quantitative Replacement of Cetyl Trimethylammonium Bromide by Cationic Thiol Ligands on the Surface of Gold Nanorods and Their Extremely Large Uptake by Cancer Cells. Angew. Chem. Int. Ed..

